# CIVIT dataset: Integral microscopy with Fourier plane recording

**DOI:** 10.1016/j.dib.2022.108819

**Published:** 2022-12-12

**Authors:** Sergio Moreschini, Filipe Gama, Robert Bregovic, Atanas Gotchev

**Affiliations:** Tampere University, Korkeakoulunkatu 1, 33720 Tampere, Finland

**Keywords:** Fourier lightfield microscopy, Light field, Blender, Z-scan, Z-stack, Cells, Filaments, DCR, Dynamic Cutting Region, DSFL, Densely Sampled Light Field, EI, Elemental Image, FiMic, Fourier Integral Microscope, FLMic, Fourier Lightfield Microscope, GT, Ground Truth, LF, Light Field, NA, Numerical Apperture, RBC, Red Blood Cell, RoI, Region of Interest

## Abstract

This article describes a dataset of synthetic images representing biological scenery as captured by a Fourier Lightfield Microscope (FLMic). It includes 22,416 images related to eight scenes composed of 3D models of objects typical for biological samples, such as red blood cells and bacteria, and categorized into Cells and Filaments groups. For each scene, two types of image data structures are provided: 51 × 51 Elemental Images (EIs) representing Densely Sampled Light Fields (DSLF) and 201 images composing Z-Scans of the scenes. Auxiliary data also includes information about camera intrinsic and extrinsic calibration parameters, object descriptions, and MATLAB scripts for camera pose compensation. The images have been generated using Blender. The dataset can be used to develop and assess methods for volumetric reconstruction from Light Field (LF) images captured by a FLMic.

**Specifications Table**

**Table:** 

Subject	Computer Vision and Pattern Recognition, Medical Imaging, Optics
Specific subject area	Light Field Microscopy
Type of data	Image Bin file 3D Model MATLAB script Blender file
How the data were acquired	Images of synthetic scenes and corresponding Z-Scans generated using 3D models in Blender. Camera parameters exported from Blender in .bin files. MATLAB script used to compensate camera poses due to limitations of Blender. Instruments: Software Blender, MATLAB
Data format	Raw
Description of data collection	8 different 3D scenes have been rendered in Blender [2]. Each scene is composed of two parts: Densely Sampled Light Field (DSLF) and Z-Scan. For each DSLF 51 × 51 images have been rendered and for each Z-Scan 201 images have been rendered, for a total of 22,416 images. All scenes were rendered using the same camera poses. Camera parameters, intrinsic and extrinsic were exported during the DSLF rendering process in an external .bin file.
Data source location	Institution: Tampere University City/Town/Region: Tampere Country: Finland
Data accessibility	Repository name: Mendeley Data Data identification number: 10.17632/22z5yhn928.2 Direct URL to data: http://dx.doi.org/10.17632/22z5yhn928.2
Related research article	S. Moreschini, R. Bregovic, A. Gotchev, Volumetric segmentation for integral microscopy with Fourier plane recording in Proc. IS&T Int'l. Symp. on Electronic Imaging: Image Processing: Algorithms and Systems, 2022, pp 356-1 - 356-6 [1]. https://doi.org/10.2352/EI.2022.34.10.IPAS-356

## Value of the Data

• The capture system simulated in Blender is a faithful reproduction of an integral microscope with Fourier plane recording: the Fourier Lightfield Microscope (FLMic), also referred to as Fourier Integral Microscope (FiMic) [3,4]. The physical FLMic outputs EIs, which form a specific coarse sampling of the 4D LF. The simulated setting allows rendering the Ground Truth (GT) in terms of DSLF and scene Z-scans and thus facilitates developing and assessing LF microscopy methods, which is attractive for its capabilities to provide 3D information from single-shot low-light dose imagery.
• Specifically, the data can be used to train deep learning models for volumetric reconstruction of microscopy specimens starting from the FLMic-captured EIs [1]. This is to appeal to researchers aiming at coupling the power of machine learning with the benefits of LF microscopy.
• In addition to images, the data includes information related to camera intrinsic and extrinsic calibration parameters (.bin), files describing the objects contained in the scenes (.obj), MATLAB scripts (.m), and a rendering example in blender (.blend) in order to allow creation of additional datasets.

## 1 Data Description

The dataset is available at Mendeley Data [5]. In the dataset, there are eight scenes recreated from 3D models in Blender, divided into two categories: Filaments and Cells. For each scene, the following data is provided:

1. RAW DSLF captured by a moving orthographic camera. A DSLF is defined as the LF having a maximal disparity between consecutive views less than 1 pixel [6]. Each DSLF is composed of 2,601 images captured on a regular grid of 51 × 51 positions – see Fig. 1(a).

**Fig. 1:**
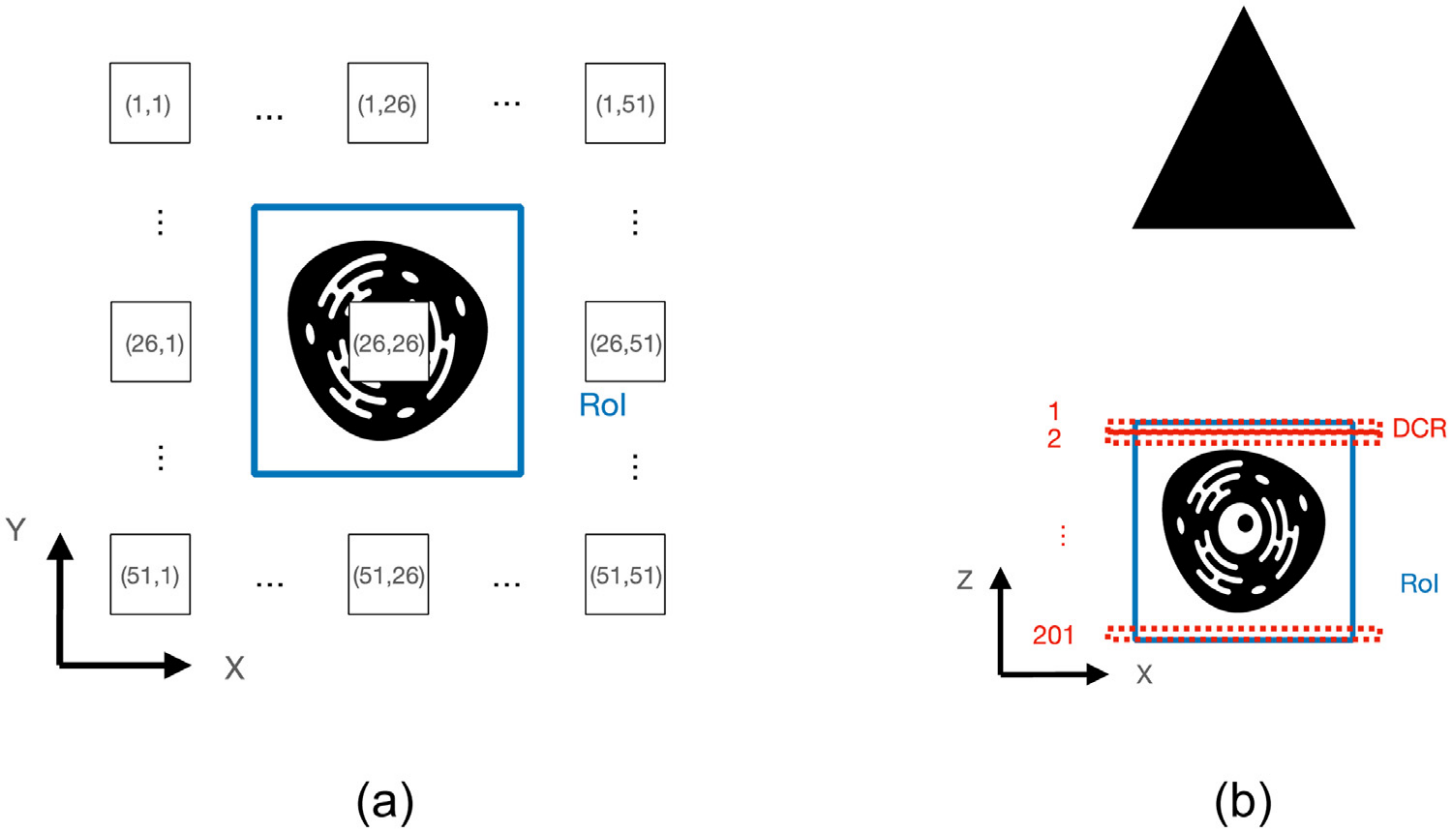
Infographics: (a) DSLF. (b) Z-Scan.
2. Z-Scan captured by an orthographic camera. A Z-Scan, often also referred to as Z-Stack, is a sequence of images, where each image represents a section of the specimen perpendicular to the optical axis denoted as the z-axis, and at a specific depth along the same axis [7]. Each Z-Scan is composed of 201 images which are bounded in a specific region defined as Region of Interest (RoI) – see Fig. 1(b).
3. ’camInfo.bin’ file composed of a header with 5 entries: Resolution on the X axis, Resolution on the Y axis, Orthographic scale (i.e. maximum dimension, in scene units, of the portion of space captured by the camera), number of images taken horizontally, and number of images taken vertically. After the header, for each camera, a transformation matrix (4 × 4) is given. The matrix contains the rotation matrix R (3 × 3), the translation vector T (1 × 3), and a (4 × 1) vector to enable homogeneous coordinates. To change the coordinate to the one used in MATLAB, we have multiplied the transformation matrix by an orthonormal basis. Thus (X,Y,Z) in Blender maps to (X,-Y,-Z) in MATLAB. An example of the entries for the file ‘camInfo.bin’ is given in Table 1.

**Table 1:** Samples of data in 'camInfo.bin' (Starting ten lines).

Resolution on X axis	2996			
Resolution on Y axis	2996			
Orthographic scale	7.49			
#pictures taken horizontally	51			
#pictures taken vertically	51			

Comment	#HeaderEnd			

Rotation (3 × 3)	0.9985	0.0000	-0.0547	0.0042
and	0.0030	-0.9985	0.0547	0.0042
Translation elements (1 × 3)	-0.0546	-0.0547	-0.9970	2.82111

Internal operations(4 × 1)	0.0000	0.0000	0.0000	1.0000
4. ‘camPos.bin’ file composed of a header similar to ’camInfo.bin’ file and, for each camera, 3 entries which are the (X, Y, Z) camera coordinates. An example of the entries for the file ‘camPos.bin’ is given in Table 2.

**Table 2:** Samples of data in 'camPos.bin' (starting nine lines).

Resolution on X axis	2996
Resolution on Y axis	2996
Orthographic scale	7.49
#pictures taken horizontally	51
#pictures taken vertically	51

Comment	#HeaderEnd

Coordinate X	0.1500
Coordinate Y	-0.1500
Coordinate Z	2.8131
5. 3D models available as WaveFront Objects (.obj) files.

In addition, an example in blender (.blend) and MATLAB script (.m) for generating the DSLF from the images rendered in Blender and the BIN files are provided.

**Fig. 2:**
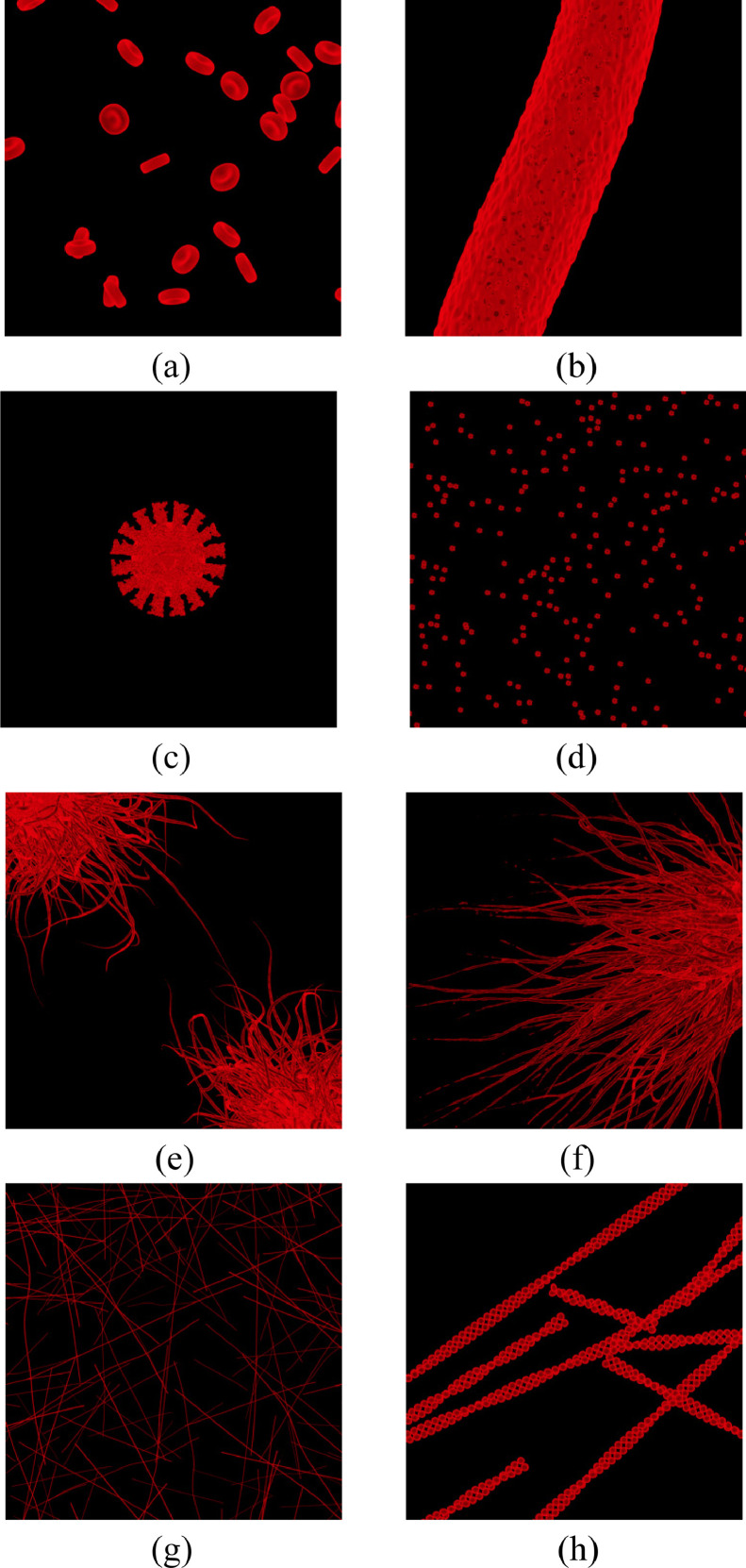
Central view for each scene composing the dataset. (a) RBC. (b) Vessel. (c) Korona. (d) Korona Particles. (e) Pili. (f) Bacteria. (g) Polymers. (h) Actin filaments.^1^

The scenes in the dataset are captured simulating monochromatic fluorescence and entitled as follows: RBC, Vessel, Korona, Korona Particles, Pili, Bacteria, Polymers and Actin. The first four compose the category Cells while the next four compose the category Filaments. The central views of each scene are shown in Fig. 2.

• **RBC**: The scene in Fig. 2(a) represents an agglomeration of Red Blood Cells (RBCs), a very common scenery in microscopy. It is composed of multiple objects with a medium thickness structure.
• **Vessel**: The scene in Fig. 2(b) represents a Vessel with RBCs seen inside it. It is composed of multiple objects nested in a single structure. Also this is a very common scenery in microscopy.
• **Korona**: The scene in Fig. 2(c) represents a high-resolution SARS-CoV spike structure. It is composed of a spherical central object with multiple objects on top of it representing the spikes. The scene has been inspired by [8].
• **Korona Particles**: The scene in Fig. 2(d) is an agglomeration of low-definition SARS- CoV spike structures. It is composed of multiple small objects.
• **Pili**: The scene in Fig. 2(e) is composed of two bacteria with Pili. The focus is on the Pili which are hair-like appendages found on the surface of various bacteria.
• **Bacteria**: The scene depicted in Fig. 2(f) is a single bacterium with Pili. Similar to Pili, it is a single body (thick structure) with multiple hair-like appendages (thin structures).
• **Polymers**: The scene in Fig. 2(g) is composed of multiple thin non-overlapping objects. The objects have different sizes and directions, and they are randomly spaced in the RoI.
• **Actin filaments**: The scene in Fig. 2(h) is composed of multiple Actin Filaments (or filamentous actin, F-actin), an essential part of the cytoskeleton [9]. The objects are composed of multiple linked smaller objects.

## 2 Experimental Design, Materials and Methods

The dataset is composed of two different kinds of representations: the DSLF and the Z-Scan.

The DSLF representation of each scene includes 51 × 51 images taken by an orthographic camera at different locations. Such camera has an orthographic scale factor of 7.49 and the sensor width is equal to 2.049 mm. All the RAW images are captured at resolution 2.996 × 2.996 pixels. After capture, the images are rotated, cropped and downsampled resulting in the final resolution of 683 × 683 pixels. During the capture stage, cameras are facing the center of the scene. The captured images are then corrected by post processing (i.e. via the provided MATLAB script). To ensure that all cameras are always facing the center of the RoI, a spherical object has been placed in the center of the RoI. For each camera, we added an Object constraint / Track to function to ensure that each camera is constantly pointing at that sphere. During rendering, the sphere was disabled to prevent its appearance in the rendered views. After capture, each scene and its related BIN files are processed through a MATLAB script “CorrectImages.m”. The MATLAB script transforms each converging camera optical axis, initially pointing towards the center of the RoI, onto parallel optical axes (i.e., parallel optical axes that are perpendicular to cameras’ baseline). At the beginning, each camera is rotated by the inverse of its rotation matrix in order to align the local camera coordinate system with the world coordinate system. Then, the original data is interpolated to the new image grid. These new images recreate the parallax effect of a FLMic [3]. The DSLF therefore represents a valid testbed for developing LF reconstruction methods, based on desired decimation and subsequent reconstruction or missing views to be compared with the GT.

Since blender is an ‘ideal’ environment for rendering, when generating the orthographic images, the limitations in resolution imposed by the effective numerical aperture (NA) of the microscope objective were not taken into account. Nevertheless, resolution-wise the dataset is realistic since the resolution of images in the dataset was selected such that it approximately corresponds to the one achievable by the physical setup described in [3]. Moreover, the aim of providing DSLF on a rectangular grid instead of elemental images for a particular micro lense setup was to make the provided dataset more generic. Particular setup of elemental images, e.g. 2-3-2 [3], 4-5-4-5 [10], 3-4-5-4-3 [11], can be easily obtained from the provided DLSF datasets by averaging over views in the DSLF that fall in each aperture of the desired micro lens setup.

The Z-Scan representation includes 201 slices of the scene. Such slices have been rendered in Blender by using a dynamic cutting region (DCR). The capture stage has been performed by using the central view of the 51 × 51 DSLF. The DCR linearly moves from a starting point, which is equivalent to the beginning on the RoI in frame 1, and reaches the ending point in frame 201 which is equivalent to the end of the RoI (Fig. 1). By making use of an external Blender tool, Jmesh [12], a dynamic Boolean is applied such that only the objects which are included in the DCR are rendered during the “Render Animation” process. We make use of the Z-Scan representation to perform image reconstruction as in optical microscopy. In order to make the Z-Scan compatible with the same dimensions of the DSLF, each rendered image is cropped both horizontally and vertically by 66 pixels and then resized by a factor of 4. All images generated through Blender have been rendered using Cycles [2].

The provided Z-Scan images represent ideal sectioning, that is, they ignore issues in optical sectioning that would occur in a physical microscope, e.g. overlap between adjacent section, contributions of objects outside of the focal plane (out-of-focus light). Using the ideal Z-scan images and knowing the model of a particular physical microscope used for optical sectioning, those effects could be added to the provided Z-scan.

## Ethics Statement

The authors declare that the work described in this paper is original, and all authors contributed to and approved the publication. The dataset does not include animals, human subjects, or data collected from social media. There is no conflict of interest in this submission.

## CRediT Author Statement

**Sergio Moreschini:** Conceptualization, Methodology, Software, Writing – original draft preparation; **Filipe Gama:** Data curation, Methodology, Investigation, Writing – review & editing; **Robert Bregovic:** Visualization, Investigation, Validation, Writing – review & editing, Supervision; **Atanas Gotchev:** Validation, Writing – review & editing, Supervision.

## Declaration of Competing Interest

The authors declare that they have no known competing financial interests or personal relationships that could have appeared to influence the work reported in this paper.

## Data Availability

CIVIT Dataset: Integral Microscopy with Fourier Plane Recording (Original data) (Mendeley Data).

## Footnotes

1 Figures 2(a), 2(d) and 2(e) reprinted from [1] with permission of IS&T: The Society for Imaging Science and Technology sole copyright owners of the Electronic Imaging: Image Processing: Algorithms and System XX
